# Relationship between cardiac biomarker concentrations and long-term mortality in subjects with osteoarthritis

**DOI:** 10.1371/journal.pone.0242814

**Published:** 2020-12-02

**Authors:** Martin Rehm, Gisela Büchele, Raphael Simon Peter, Rolf Erwin Brenner, Klaus-Peter Günther, Hermann Brenner, Wolfgang Koenig, Dietrich Rothenbacher

**Affiliations:** 1 Institute of Epidemiology and Medical Biometry, Ulm University, Ulm, Germany; 2 Division for Biochemistry of Joint and Connective Tissue Diseases, Department of Orthopedics, Ulm University, Ulm, Germany; 3 University Center of Orthopedic and Trauma Surgery, Technical University of Dresden, Dresden, Germany; 4 Division of Clinical Epidemiology and Aging Research, German Cancer Research Center (DKFZ), Heidelberg, Germany; 5 Division of Preventive Oncology, German Cancer Research Center (DKFZ) and National Center for Tumor Diseases (NCT), Heidelberg, Germany; 6 German Cancer Consortium (DKTK), German Cancer Research Center (DKFZ), Heidelberg, Germany; 7 Deutsches Herzzentrum München, Technische Universität München, Munich, Germany; 8 German Centre for Cardiovascular Research (DZHK), Partner Site Munich Heart Alliance, Munich, Germany; Ospedale del Cuore G Pasquinucci Fondazione Toscana Gabriele Monasterio di Massa, ITALY

## Abstract

Osteoarthritis (OA) is associated with adverse cardio-metabolic features. N-terminal pro-B-type natriuretic peptide (NT-proBNP) and high-sensitivity troponins T and I (hs-cTnT and hs-cTnI) are well-characterized cardiac markers and provide prognostic information. The objective was to assess the association of cardiac biomarker concentrations with long-term mortality in subjects with OA. In a cohort of 679 OA subjects, undergoing hip or knee replacement during 1995/1996, cardiac biomarkers were measured and subjects were followed over 20 years. During a median follow-up of 18.4 years, 332 (48.9%) subjects died. Median of hs-cTnT, hs-cTnI, and NT-proBNP at baseline was 3.2 ng/L, 3.9 ng/L, and 96.8 ng/L. The top quartile of NT-proBNP was associated with increased risk of mortality (Hazard Ratio (HR) 1.79, 95% confidence interval (CI) 1.17–2.73) after adjustment for covariates including troponins (hs-cTnT HR 1.30 (95% CI 0.90–1.89), hs-cTnI HR 1.32 (95% CI 0.87–2.00) for top category). When biomarker associations were evaluated as continuous variables, only NT-proBNP (HR per log-unit increment 1.34, 95% CI 1.16–1.54) and hs-cTnI (HR 1.38, 95% CI 1.11–1.72) showed robust results. Elevated cardiac biomarker concentrations predicted an increased risk of long-term mortality and strongest for NT-proBNP and hs-cTnI. These results might help to identify subjects at risk and target preventive efforts early.

## Introduction

Cardiac troponins are established biomarkers for the diagnosis of myocardial infarction (MI) [1]. Moreover, the use of highly sensitive assays allows the measurement of serum concentrations well below the diagnostic threshold for MI. Serum levels in asymptomatic populations are associated with an increased risk of subsequent cardiovascular and total mortality [2, 3].

Osteoarthritis (OA) is a common age-related degenerative disease of the musculoskeletal system that causes severe joint pain and loss of function [4]. In addition to determinants such as genetic predisposition, advanced age, female sex, joint injury, and increased body mass, various systemic etiological conditions such as inflammation, inflamed adipose tissue, and dyslipidemia are known to be risk factors for OA [5–9]. Furthermore, it has been shown that the presence of metabolic syndrome determines the overall risk for the development and progression of OA. Especially the relationship between obesity and metabolic syndrome in OA has become increasingly discernible [10]. Therefore, OA and cardiovascular phenotypes may be closely related and may also share common pathophysiological pathways [7, 8]. So far, the role of well-established cardiovascular biomarkers in a population with OA has not been evaluated.

This study aimed to assess associations of biomarker concentrations of cardiac damage (i.e., high-sensitivity cardiac troponins T and I (hs-cTnT and hs-cTnI)) and myocardial hemodynamic stress (N-terminal pro-B-type natriuretic peptide (NT-proBNP)) with long-term mortality in subjects with hip or knee OA after consideration of other established risk factors and covariates.

## Materials and methods

### Study population

The Ulm Osteoarthritis Study is a multi-center cohort study of subjects who underwent unilateral total hip or knee replacement due to advanced OA between January 1995 and December 1996. All subjects were Caucasians and did not exceed the age of 75 years at baseline. Subjects with malignancies, inflammatory diseases, corticosteroid medication, or previous hip or knee joint replacement were excluded. In total, 809 eligible subjects provided written informed consent and underwent baseline assessments in Ulm, Augsburg, or Stuttgart (three cities in the South of Germany). Further details have been described previously [11, 12].

For this study, those with missing information for biomarker concentrations at baseline (n = 130) were excluded. Thus, 679 subjects remained for this analysis. The study and the current follow-up were approved by the Ethics Committee of Ulm University and were conducted in accordance with the relevant guidelines and regulations.

### Data collection and laboratory methods

Baseline demographic data (e.g., age, sex, weight, height, and smoking status) and information on self-reported medical history (e.g., diabetes mellitus, hypertension, myocardial infarction, and heart failure) were collected by standardized questionnaires in personal interviews. Blood at baseline was drawn preoperatively under standardized conditions, centrifuged, aliquoted, and stored at -80°C until further analysis.

Cardiac biomarkers and cystatin C were measured in frozen non-fasting serum samples in the year 2018. High-sensitivity cardiac troponin T (hs-cTnT) concentrations were measured by the Elecsys Troponin T hs Test (Roche Diagnostics) with a limit of blank (LoB) of 3.0 ng/L, a limit of detection (LoD) of 5.0 ng/L, and inter-assay coefficients of variation (CVs) of 3.6% and 2.9% at concentrations of 42.0 and 2.8 ng/L, respectively. High-sensitivity cardiac troponin I (hs-cTnI) concentrations were measured with the Architect STAT High Sensitivity Troponin I Test (Abbott Diagnostics) with an LoD of 2.0 ng/L and inter-assay CVs between 5.9 and 6.7 ng/L at three different concentrations, and finally, NT-proBNP levels were determined on an Elecsys assay (Roche Diagnostics) with an LoD of 5.0 ng/L.

Serum cystatin C was measured by immunonephelometry on a BNA II (Behring Werke, Marburg, Germany) with an inter-assay CV between 2.9 and 3.2%. High-sensitivity C-reactive protein (hs-CRP) was measured by the nephelometric method using a commercial kit (NA-latex CRP, Behring Werke, Marburg, Germany) calibrated with the WHO reference standard 85/506 [7].

### Mortality follow-up

The primary endpoint of this evaluation was all-cause mortality, determined up to 20 years after joint replacement. The registration authorities were contacted to obtain the survival status and the exact date of death in case the subject died. Further details can also be found elsewhere [13].

The follow-up time was calculated in days, starting with the date of the baseline interview and ending with the date of death. Non-deceased subjects were censored at the time they were last known to be alive (the latest date of censoring was September 22, 2015).

### Statistical analysis

The study population was described in terms of various demographic and medical characteristics. Categorical variables were represented as proportions and continuous variables as medians with interquartile ranges. Associations of demographics, various cardiovascular risk factors, and biomarker distributions were quantified using the nonparametric Kruskal-Wallis test.

Partial Spearman correlation coefficients, adjusted for age and sex, were calculated to quantify the correlation between various laboratory measures, including the cardiac biomarkers. The biomarker concentrations were natural log-transformed for further analyses because of their skewed distribution.

Adjusted hazard ratios (HR) with 95% confidence intervals (CI) were estimated using multivariable Cox proportional hazards models to assess the association of cardiac biomarker concentrations (classified in four categories and continuously after log-transformation) with long-term mortality. NT-proBNP and hs-cTnI were categorized by quartiles. For hs-cTnT, the reference category contains those subjects with concentrations below 3.0 ng/L (LoB) (as suggested in a recent meta-analysis by Parikh et al. [14]), with the remainder of the sample divided into three equal parts. A total of 323 subjects (47.6%) showed hs-cTnT <3.0 ng/L and 2 subjects (0.3%) showed NT-proBNP <5.0 ng/L. These measurements were set to 1.5 ng/L (half of the LoB of the hs-cTnT assay) and 2.5 ng/L (half of the LoD of the NT-proBNP assay), respectively.

Different sets of adjustment variables have been defined a priori. The proportional hazards assumption was evaluated graphically and based on a multivariable model (Model 3 in Table 3). In Model 1, hazard ratios were adjusted for age (continuous) and sex (male, female). In Model 2, the analysis was also adjusted for established clinical risk factors, i.e., the body-mass index (continuous), the smoking status (never, former, current), the localization of the OA (hip, knee), the history of diabetes mellitus (yes, no), serum cholesterol (continuous), and the continuous log-transformed cystatin C concentrations. In Model 3, the analysis was adjusted for Model 2 covariates and simultaneously for continuous log-transformed cardiac biomarker concentrations.

Areas under the receiver-operating characteristic (ROC) curve (AUC) were calculated to assess the discriminative capacity of hs-cTnT, hs-cTnI, and NT-proBNP for mortality. The net reclassification improvement (NRI) in cases and non-cases calculated by the Kaplan-Meier estimator was used to examine the predictive value of hs-cTnT, hs-cTnI, and NT-proBNP according to the 1%, 5%, and 10% risk strata of the predicted probability for death after ten years [15]. The bootstrap method was used to create 95% confidence intervals for the NRI estimates [16]. Besides, dose-response relationships between cardiac biomarker levels and survival outcomes were plotted with restricted cubic splines using four knots and the median concentration in the lowest category as the reference.

Statistical analysis was performed using SAS version 9.4 (SAS Institute Inc., Cary, NC, USA) and R version 3.5.1 (The R Foundation for Statistical Computing, Vienna, Austria).

## Results

### Subject characteristics

**Table 1** summarizes the baseline demographic characteristics of the 679 subjects included in this analysis (median age 65 years, 63% female) with hip (53%) or knee (47%) OA. More than three out of four subjects (77.3%) had a body mass index (BMI) of 25 kg/m^2^ or more, and almost half of the subjects (42%) were former or current smokers. A history of diabetes mellitus, hypertension, myocardial infarction, and heart failure was reported by 8.7%, 50.5%, 4%, and 18.7% of subjects, respectively.

**Table 1:** Baseline characteristics and median biomarker concentrations.

	Total	Median	Median	Median
	(N = 679)	hs-cTnT,	hs-cTnI,	NT-proBNP,
		ng/L	ng/L	ng/L
Age, years	65.0 (58.0,70.0)			
<60 years	196 (28.9)	<3.00	3.00	53.03
≥60 to <70 years	296 (43.6)	3.38	4.45	115.30
≥70 years	187 (27.5)	5.30	4.50	126.00
Sex				
Male	251 (37.0)	3.89	4.10	69.79
Female	428 (63.0)	<3.00	3.80	113.20
Osteoarthritis localization				
Hip	360 (53.0)	<3.00	3.50	85.67
Knee	319 (47.0)	3.73	4.40	104.30
Body mass index, kg/m^2^	27.8 (25.3,30.8)			
<25 kg/m^2^	154 (22.7)	<3.00	3.20	99.97
≥25 to <30 kg/m^2^	322 (47.4)	3.22	4.10	95.43
≥30 to <35 kg/m^2^	160 (23.6)	3.86	4.40	101.95
≥35 kg/m^2^	43 (6.3)	3.73	4.60	99.34
Smoking status				
Never	394 (58.0)	3.11	3.90	103.55
Former	200 (29.5)	3.81	3.90	95.01
Current	85 (12.5)	<3.00	3.70	67.50
History of diabetes mellitus				
No	620 (91.3)	3.05	3.90	96.45
Yes	59 (8.7)	5.52	4.90	101.70
History of hypertension				
No	336 (49.5)	<3.00	3.10	79.24
Yes	343 (50.5)	4.12	4.70	117.30
History of myocardial infarction				
No	652 (96.0)	3.17	3.90	96.67
Yes	27 (4.0)	6.42	5.50	136.60
History of heart failure				
No	552 (81.3)	<3.00	3.70	90.00
Yes	127 (18.7)	5.61	5.20	143.60

Values are median (first quartile, third quartile) or n (percentage).

### Serum concentrations of cardiac biomarkers

As further depicted in **Table 1**, the median concentrations of hs-cTnT, hs-cTnI, and NT-proBNP were associated with increasing age and were higher in subjects having a history of hypertension, myocardial infarction, or heart failure. Cardiac biomarker concentrations were also higher in subjects with knee OA. However, the higher age of these subjects compared to hip OA must be taken into account (details in [13]). Furthermore, subjects with primary causes of OA showed higher median levels of hs-cTnT, hs-cTnI, and NT-proBNP (3.51 ng/L, 4.20 ng/L, and 104.50 ng/L, respectively) in comparison to secondary OA (<3.00 ng/L, 3.40 ng/L, and 76.14 ng/L, respectively) (data not in the table). Female subjects showed lower hs-cTnT and hs-cTnI values, but higher levels of NT-proBNP. Subjects with diabetes had higher levels of hs-cTnT and hs-cTnI compared to non-diabetics.

**Table 2** shows the values of laboratory parameters and their age- and sex-adjusted partial Spearmen rank correlations with cardiac biomarkers. The median level (first quartile–third quartile) of hs-cTnT, hs-cTnI, and NT-proBNP at baseline was 3.2 (1.5–6.2) ng/L, 3.9 (2.8–5.7) ng/L, and 96.8 (52.7–182.6) ng/L, respectively. The cardiac troponins and NT-proBNP, as well as cystatin C (0.9 (0.8–1.0) mg/L), were positively correlated with each other. The strongest correlation was seen between both troponins (Rho = 0.40). The correlation of NT-proBNP with hs-cTnT was 0.24 while with hs-cTnI, it was 0.35 (both p-values <0.001).

**Table 2:** Partial Spearman rank correlation coefficients.

		hs-cTnT		hs-cTnI		NT-proBNP	
	Median (Q1,Q3)	Rho	p	Rho	p	Rho	p
hs-cTnT, ng/L	3.2 (1.5,6.2)						
hs-cTnI, ng/L	3.9 (2.8,5.7)	0.40	< .001				
NT-proBNP, ng/L	96.8 (52.7,182.6)	0.24	< .001	0.35	< .001		
Cystatin C, mg/L	0.9 (0.8,1.0)	0.21	< .001	0.18	< .001	0.19	< .001
hs-CRP, mg/L	2.6 (1.3,5.0)	0.07	0.066	0.14	< .001	0.05	0.204
Cholesterol, mmol/L	5.7 (5.1,6.4)	-0.05	0.245	0.08	0.042	-0.02	0.581

Correlation coefficients (Rho) and p-values are adjusted for age and sex. Q1 = first quartile. Q3 = third quartile.

N = 97 values are missing for cholesterol.

### Serum cardiac biomarker levels and mortality prediction

The association of hs-cTnT, hs-cTnI, and NT-proBNP as analyzed in quartiles with total mortality is depicted in **Table 3**. Over a median follow-up period of 18.4 years, 332 (48.9%) subjects died. The mortality rate was 32.7 deaths per 1000 person-years. After adjustment for age and sex, the top categories of the biomarker concentrations were all associated with increased mortality compared to the bottom categories (hs-cTnT: Hazard Ratio (HR) 2.07, 95% confidence interval (CI) 1.55–2.77, hs-cTnI: HR 2.23 (95% CI 1.58–3.16), NT-proBNP: HR 2.50 (95% CI 1.74–3.60)) and there was always a statistically significant p-value for the trend across categories. This association also persisted after further adjustment for several established covariates, i.e., BMI, smoking status, localization of OA, diabetes mellitus, cholesterol, and cystatin C (hs-cTnT: HR 1.81 (95% CI 1.29–2.55), hs-cTnI: HR 1.98 (95% CI 1.35–2.88), NT-proBNP: HR 2.30 (95% CI 1.53–3.44)) with statistically significant p-values for the trend. However, after simultaneous adjustment for the cardiac biomarkers (Model 3), a statistically significant relationship for hs-cTnT was lost. Nevertheless, it was still present for hs-cTnI (p-value for trend = 0.017; HR per log unit increase 1.38 (95% CI 1.11–1.72)) and strongest for NT-proBNP: HR 1.79 (95% CI 1.17–2.73) when top quartiles were compared to bottom ones (p-value for trend = 0.013; HR per log unit increase 1.34 (95% CI 1.16–1.54)). Notably, the HRs did not change substantially with increasing follow-up time (**S1 Fig**). **S1 Table** shows the analyses in tertiles.

**Table 3:** Cox proportional regression analysis for mortality.

	Events/N	Rate per 1000 p-yr	Model 1^a^	Model 2^b^	Model 3^c^
			HR (95% CI)	HR (95% CI)	HR (95% CI)
hs-cTnT, ng/L					
<3.00	106/323	20.5	1.00	1.00	1.00
3.00–4.83	66/118	36.9	1.32 (0.97–1.81)	1.45 (1.03–2.04)	1.27 (0.90–1.80)
4.84–7.73	66/118	39.4	1.26 (0.92–1.72)	1.22 (0.86–1.73)	1.05 (0.74–1.50)
>7.73	94/120	62.0	2.07 (1.55–2.77)	1.81 (1.29–2.55)	1.30 (0.90–1.89)
p for trend			< .001	0.003	0.305
Per unit increase^d^	332/679	32.7	1.37 (1.22–1.55)	1.32 (1.15–1.51)	1.15 (0.98–1.34)
hs-cTnI, ng/L					
<2.90	47/176	16.3	1.00	1.00	1.00
2.90–3.90	65/168	24.5	0.96 (0.65–1.41)	0.93 (0.62–1.41)	0.80 (0.53–1.22)
4.00–5.60	99/161	41.6	1.57 (1.10–2.25)	1.61 (1.09–2.36)	1.29 (0.87–1.92)
>5.60	121/174	54.2	2.23 (1.58–3.16)	1.98 (1.35–2.88)	1.32 (0.87–2.00)
p for trend			< .001	< .001	0.017
Per unit increase^d^	332/679	32.7	1.79 (1.53–2.09)	1.76 (1.47–2.11)	1.38 (1.11–1.72)
NT-proBNP, ng/L					
<52.74	46/169	16.5	1.00	1.00	1.00
52.74–96.68	78/170	30.2	1.52 (1.05–2.21)	1.41 (0.93–2.13)	1.34 (0.89–2.03)
96.81–182.10	86/170	32.7	1.43 (0.98–2.08)	1.34 (0.88–2.02)	1.21 (0.80–1.83)
>182.10	122/170	56.9	2.50 (1.74–3.60)	2.30 (1.53–3.44)	1.79 (1.17–2.73)
p for trend			< .001	< .001	0.013
Per unit increase^d^	332/679	32.7	1.52 (1.36–1.70)	1.51 (1.32–1.72)	1.34 (1.16–1.54)

N = number of observed subjects. p-yr = person-years. HR = hazard ratio. CI = confidence interval.

^a^Adjusted for age and sex.

^b^Adjusted for age, sex, BMI, smoking status, localization of OA, diabetes, cholesterol, and log-transformed concentration of cystatin C.

^c^Adjusted for age, sex, BMI, smoking status, localization of OA, diabetes, cholesterol, and log-transformed concentrations of cystatin C and the other two cardiac biomarkers (i.e. in case of hs-cTnT then hs-cTnI and NT-proBNP, in case of hs-cTnI then hs-cTnT and NT-proBNP and in case of NT-proBNP then hs-cTnT and hs-cTnI, respectively).

^d^After logarithmic transformation.

Measures of model discrimination and reclassification related to the models of Table 3 are shown in **Table 4**. A small increase was seen in the area under the ROC curve from 0.73 (95% CI, 0.70–0.76) to 0.75 (95% CI, 0.72–0.78) when hs-cTnT, hs-cTnI, and NT-proBNP were added to the basic model. While the net reclassification index in the deceased (event NRI) was only slightly different from zero, the positive non-event NRI of 0.11 (95% CI, 0.04–0.22) indicates that adding the biomarkers of interest to the model resulted in more subjects being classified into lower-risk categories among those without event (increase in specificity).

**Table 4:** Measures of model discrimination and reclassification.

	AUC (95% CI)	NRI_e_ (95% CI)	NRI_ne_ (95% CI)
Basic model^a^	0.73 (0.70–0.76)		
Basic model^a^ + ln(hs-cTnT)	0.74 (0.71–0.77)	0.03 (-0.07,0.08)	0.03 (-0.02,0.09)
Basic model^a^ + ln(hs-cTnI)	0.74 (0.71–0.77)	0.00 (-0.11,0.05)	0.09 (0.02,0.16)
Basic model^a^ + ln(NT-proBNP)	0.74 (0.71–0.77)	0.04 (-0.04,0.10)	0.08 (0.02,0.16)
Full model^b^	0.75 (0.72–0.78)	0.05 (-0.06,0.10)	0.11 (0.04,0.22)

NRI_e_ = Event net reclassification index; NRI_ne_ = Non-event net reclassification index.

^a^ Adjusted for age, sex, BMI, smoking status, localization of OA, diabetes, cholesterol, and log-transformed concentration of cystatin C.

^b^ Adjusted for age, sex, BMI, smoking status, localization of OA, diabetes, cholesterol, and log-transformed concentrations of cystatin C, hs-cTnT, hs-cTnI, and NT-proBNP.

**Fig 1:**
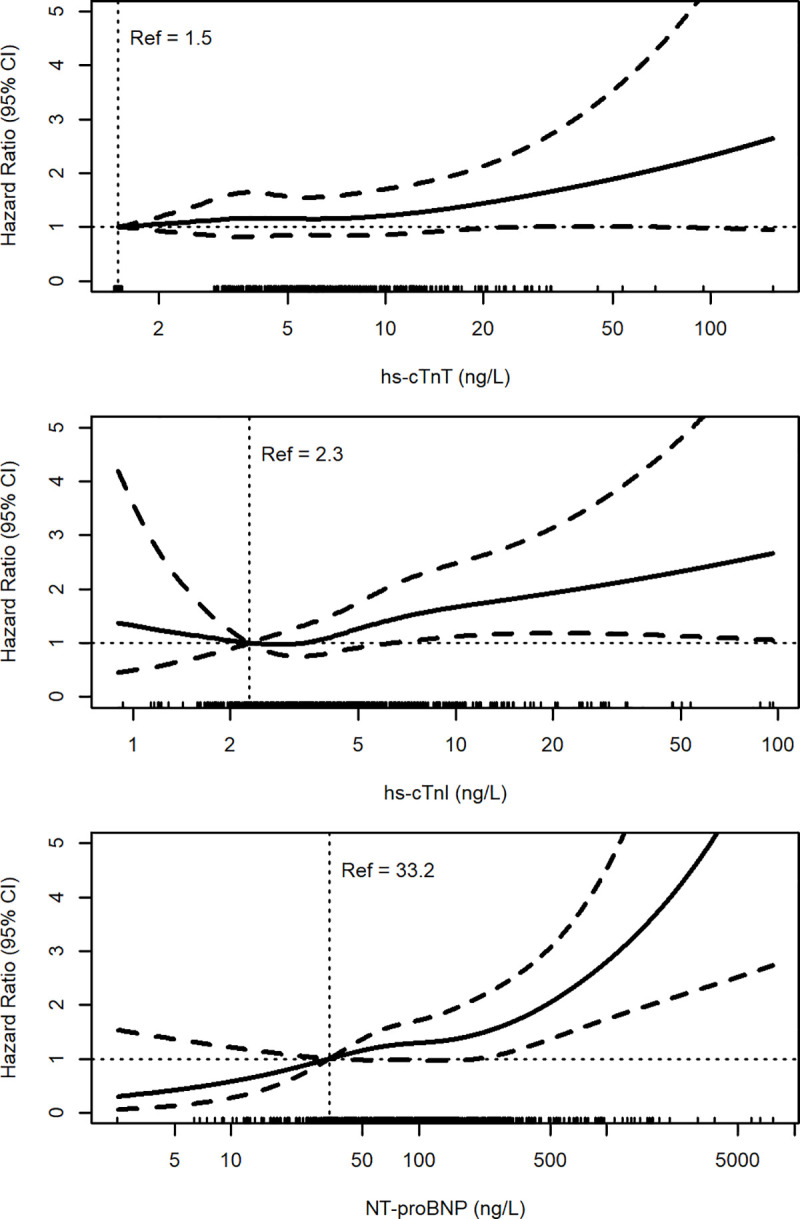
Relationship between cardiac biomarker levels and mortality. Adjusted for age, sex, BMI, smoking status, localization of OA, diabetes, cholesterol, and log-transformed concentrations of cystatin C, hs-cTnT, hs-cTnI, and NT-proBNP.

Fig 1 shows restricted cubic spline curves representing the dose-response relationship between cardiac biomarker levels (i.e., hs-cTnT, hs-cTnI, and NT-proBNP) and mortality indicating an independent statistically significant risk increase for long-term mortality associated with hs-cTnI and NT-proBNP concentrations in the simultaneously adjusted model (adjusted as Model 3 in Table 3).

## Discussion

In the present study, including 679 subjects with advanced OA and unilateral hip or knee replacement, we found robust associations of baseline hs-cTnT, hs-cTnI, and NT-proBNP measurements with all-cause mortality during long-term follow-up of 20 years. Elevated biomarker levels at baseline were associated with increased mortality after adjusting for age and sex. This effect was only slightly attenuated after further adjusting for several traditional risk factors. Although mutual adjustment of the various cardiac biomarkers led to a more pronounced weakening of the associations with mortality, associations remained statistically significant for NT-proBNP and hs-cTnI measurements.

### Prognostic value of cardiac troponins and NT-proBNP

The high-sensitivity cardiac troponins and NT-proBNP are important predictors for cardiovascular events and increased risk of death in subjects with coronary heart disease (CHD) or heart failure [17–20], but also in the general population [2, 3, 21–23]. Even slightly increased troponin levels were associated with clinically important increased mortality in a large number of routinely tested consecutive subjects from the UK, regardless of age. Notably, most of these subjects had no history of the acute coronary syndrome [24]. Parikh and colleagues also showed that slightly elevated, but still very low concentrations of hs-cTnT between 3.0 and <5.0 ng/L were associated with a higher prevalence of cardiovascular risk factors, more cardiac pathology, and worse outcomes compared to concentrations below 3.0 ng/L [14]. NT-proBNP, hs-cTnT, and hs-cTnI were also independently associated with all-cause mortality in a cohort of community-dwelling older adults from Germany [23] and cTnT and according to a meta-analysis, cTnI showed robust associations with cardiovascular and all-cause mortality in the general population [25]. To our knowledge, however, the prognostic value of these markers has not been evaluated in a population of OA subjects so far.

The results of a systematic review and meta-analysis by Hall et al. [26] suggest that subjects with OA have a significantly higher prevalence of cardiovascular disease than subjects without OA. Traditional risk factors for cardiovascular disease, including age, sex, smoking, and components of the metabolic syndrome (i.e., overweight, dyslipidemia, and diabetes mellitus), are also associated with the development and progression of OA, suggesting common pathophysiological pathways in their development. Indeed, in our study population, the highest concentrations of the cardiac biomarkers were found in elderly, obese subjects and subjects with cardiometabolic comorbidities, namely with a high cardiometabolic risk profile. Regarding inflammatory status as indicated by hs-CRP baseline serum levels, our study population was in-between a general population [27] and a population with cardiovascular disease [18].

NT-proBNP appears to have the strongest prognostic value of the three investigated markers in this cohort. This may be related to the fact that NT-proBNP is a reliable marker of left ventricular function and OA subjects are at increased risk for heart failure compared to non-arthritic subjects, as seen in a recent systematic review and meta-analysis [26]. In a supplementary analysis, in which the population was divided into tertiles of the biomarkers, a risk increase of a similar magnitude was found, and in particular, as observed in other studies[22, 28, 29], the top tertile of the hs-TnI distribution showed a significantly higher risk than the other two (S1 Table).

The results of this analysis suggest that the assessment of cardiac biomarkers may be a useful complement to traditional risk factors for predicting mortality in subjects with OA and may be used for counseling subjects or trigger specific interventions to reduce relevant cardiovascular risk factors. The present data, which showed correlations with total mortality, suggest that even slightly elevated cardiac biomarker concentrations can identify subclinical structural health impairments and contributors to the risk of premature death. In contrast to the results shown in a population with cardiovascular disease, the hazard ratios in our study (S1 Fig) did not change substantially with increasing follow-up time [30].

Two recent studies have recommended that cardiac biomarkers, and especially cardiac troponins, should be used for cardiovascular screening in the general population[31, 32]. The results of our study referring to the association of elevated cardio-specific biomarkers with high cardiovascular risk in patients with OA support these recommendations, especially for patients with OA. An important step would now be to identify the cardiometabolic risk factors associated with elevated levels of cardiac biomarkers and to determine which ones could be a target for early intervention. Evidence of successful risk factor intervention, followed by a decrease in cardiovascular biomarker levels, would then be the next step.

### Strengths and limitations

The current study population of 679 subjects was well characterized at baseline for information on established risk factors for death and cardiovascular diseases. In addition, detailed assessments of laboratory data were available. Other strengths include the long follow-up time of 20 years and the relatively high number of deaths, extending the statistical power for this outcome. Another strength is that the biomarker concentrations were measured with highly sensitive assays and high quality.

Limitations of the present study include a lack of detailed information about the long-term stability of the measured markers in frozen samples. However, in another study, we evaluated the 8-year stability of NT-proBNP serum samples stored at -80°C with a maximum of one defrost cycle and found an estimated recovery between 89.5% and 103% [33]. Furthermore, only subjects with advanced hip or knee OA, which required unilateral joint replacement were included, which involves a selection of subjects and might limit the generalizability of the results.

The variability of the distribution of biomarkers can be critical in statistical analysis using regression models [31]. In addition, within the narrow normal range, relatively small changes in concentration can lead to a classification into another risk category, which increases the importance of the measurement accuracy of the assay [32]. One measure to overcome these problems could be to confirm the values by repeated measurements and to assess longitudinal changes. However, there is evidence that serial measurements of hs-cTnI during long-term follow-up are not superior in its prognostic value compared to the most recent single measurement in the general population and that a single measurement of cardiac troponins should be sufficient to predict cardiovascular risk [34, 35].

Unfortunately, no cause-specific data on deaths, including cardiovascular mortality, were available due to German data protection regulations. However, we would expect that the associations to cardiovascular mortality are even stronger than the associations currently presented for all-cause mortality.

## Conclusions

In this cohort of subjects with OA, elevated cardiac biomarker concentrations at baseline predicted increased mortality during a 20-year follow-up. This association was independent of other risk factors and was strongest for NT-proBNP and hs-cTnI. These results could help to identify subjects at higher risk of mortality and to consider early targeting of prevention measures.

## Supporting information

S1 Fig Cox proportional regression analysis for mortality using different follow-up periods. Adjusted for age, sex, BMI, smoking status, localization of OA, diabetes, cholesterol, and log-transformed concentrations of cystatin C and the other two cardiac biomarkers (i.e. in case of hs-cTnT then hs-cTnI and NT-proBNP, in case of hs-cTnI then hs-cTnT and NT-proBNP and in case of NT-proBNP then hs-cTnT and hs-cTnI, respectively).
(TIF)

S1 Table Cox proportional regression analysis for mortality with biomarkers categorized into tertiles. N = number of observed subjects. p-yr = person-years. HR = hazard ratio. CI = confidence interval. ^a^Adjusted for age and sex. ^b^Adjusted for age, sex, BMI, smoking status, localization of OA, diabetes, cholesterol, and log-transformed concentration of cystatin C. ^c^Adjusted for age, sex, BMI, smoking status, localization of OA, diabetes, cholesterol, and log-transformed concentrations of cystatin C and the other two cardiac biomarkers (i.e. in case of hs-cTnT then hs-cTnI and NT-proBNP, in case of hs-cTnI then hs-cTnT and NT-proBNP and in case of NT-proBNP then hs-cTnT and hs-cTnI, respectively).
(DOCX)
