# Supplementary figures and images for: Relationship between cardiac biomarker concentrations and long-term mortality in subjects with osteoarthritis
Source: PLoS One. 2020 Dec 2;15(12):e0242814. doi: 10.1371/journal.pone.0242814 (PMC7710029; doi:10.1371/journal.pone.0242814)

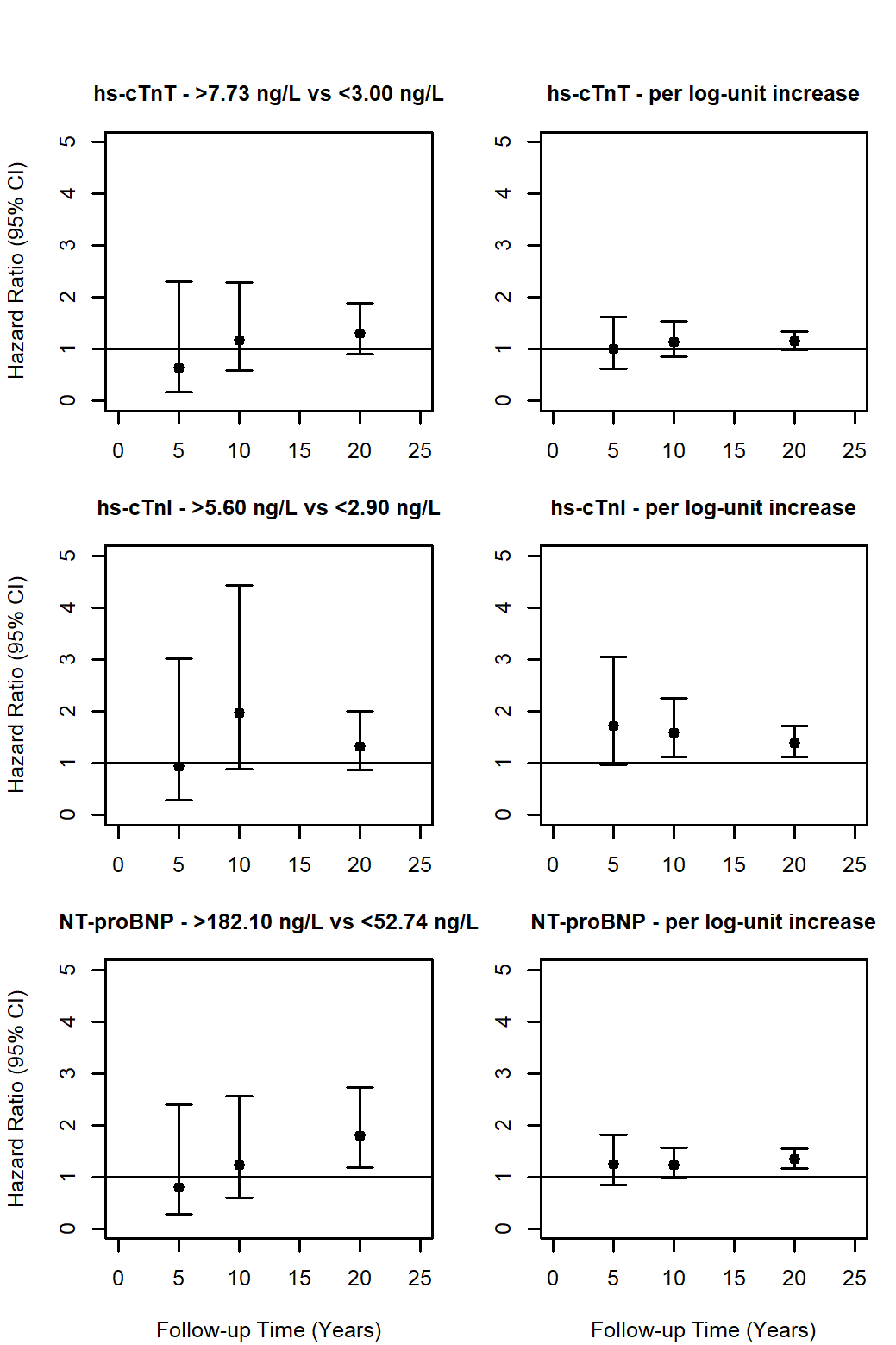

Supplement: S1 Fig — Adjusted for age, sex, BMI, smoking status, localization of OA, diabetes, cholesterol, and log-transformed concentrations of cystatin C and the other two cardiac biomarkers (i.e. in case of hs-cTnT then hs-cTnI and NT-proBNP, in case of hs-cTnI then hs-cTnT and NT-proBNP and in case of NT-proBNP then hs-cTnT and hs-cTnI, respectively). (TIF) [file pone.0242814.s001.tif]
