# Supplementary material for: Relationship between cardiac biomarker concentrations and long-term mortality in subjects with osteoarthritis
Source: PLoS One. 2020 Dec 2;15(12):e0242814. doi: 10.1371/journal.pone.0242814 (PMC7710029; doi:10.1371/journal.pone.0242814)
Supplement: S1 Table — N = number of observed subjects. p-yr = person-years. HR = hazard ratio. CI = confidence interval. aAdjusted for age and sex. bAdjusted for age, sex, BMI, smoking status, localization of OA, diabetes, cholesterol, and log-transformed concentration of cystatin C. cAdjusted for age, sex, BMI, smoking status, localization of OA, diabetes, cholesterol, and log-transformed concentrations of cystatin C and the other two cardiac biomarkers (i.e. in case of hs-cTnT then hs-cTnI and NT-proBNP, in case of hs-cTnI then hs-cTnT and NT-proBNP and in case of NT-proBNP then hs-cTnT and hs-cTnI, respectively). (DOCX) [file pone.0242814.s002.docx]

**S1 Table. Cox proportional regression analysis for mortality with biomarkers categorized into tertiles**

| **Biomarker** | **Range, ng/L** | **Events/N** | **Rate per 1000 p-yr**  **(95% CI)** | **Model 1^a^**  **HR (95% CI)** | **Model 2^b^**  **HR (95% CI)** | **Model 3^c^**  **HR (95% CI)** |
| --- | --- | --- | --- | --- | --- | --- |
| hs-cTnT | <3.00 | 106/323 | 20.5 | 1.00 (Reference) | 1.00 (Reference) | 1.00 (Reference) |
|  | 3.00-5.98 | 95/178 | 36.4 | 1.22 (0.92-1.62) | 1.26 (0.92-1.72) | 1.08 (0.79-1.49) |
|  | >5.98 | 131/178 | 55.3 | 1.85 (1.42-2.42) | 1.71 (1.26-2.32) | 1.34 (0.96-1.87) |
| hs-cTnI | <3.10 | 57/214 | 16.1 | 1.00 (Reference) | 1.00 (Reference) | 1.00 (Reference) |
|  | 3.10-4.90 | 115/235 | 31.8 | 1.33 (0.96-1.84) | 1.34 (0.94-1.89) | 1.10 (0.77-1.58) |
|  | >4.90 | 160/230 | 53.4 | 2.25 (1.65-3.08) | 2.11 (1.50-2.95) | 1.47 (1.02-2.12) |
| NT-proBNP | <65.51 | 70/226 | 18.8 | 1.00 (Reference) | 1.00 (Reference) | 1.00 (Reference) |
|  | 65.51-140.30 | 111/226 | 32.6 | 1.32 (0.97-1.79) | 1.22 (0.87-1.72) | 1.14 (0.81-1.60) |
|  | >140.30 | 151/227 | 49.9 | 1.98 (1.46-2.68) | 1.87 (1.33-2.62) | 1.55 (1.10-2.19) |

N = number of observed subjects. p-yr = person-years. HR = hazard ratio. CI = confidence interval.

^a^Adjusted for age and sex.

^b^Adjusted for age, sex, BMI, smoking status, localization of OA, diabetes, cholesterol, and log-transformed concentration of cystatin C.

^c^Adjusted for age, sex, BMI, smoking status, localization of OA, diabetes, cholesterol, and log-transformed concentrations of cystatin C and the other two cardiac biomarkers (i.e. in case of hs-cTnT then hs-cTnI and NT-proBNP, in case of hs-cTnI then hs-cTnT and NT-proBNP and in case of NT-proBNP then hs-cTnT and hs-cTnI, respectively).
